# Development of a tumor control probability model for boron neutron capture therapy of head and neck cancer

**DOI:** 10.3389/fonc.2023.1249074

**Published:** 2024-02-29

**Authors:** Fang-Ying Hsu, Yen-Wan Hsueh Liu, Tzung-Yi Lin, Ling-Wei Wang

**Affiliations:** ^1^ Institute of Nuclear Engineering and Science, National Tsing Hua University, Hsinchu, Taiwan; ^2^ Research and Development Center, Heron Neutron Medical Corporation, Zhubei, Taiwan; ^3^ Department of Heavy Particles and Radiation Oncology, Taipei Veterans General Hospital, Taipei, Taiwan; ^4^ School of Medicine, National Yang Ming Chiao Tung University, Hsinchu, Taiwan

**Keywords:** tumor control probability, boron neutron capture therapy, head and neck cancer, clinical trial, biological effective dose

## Abstract

The tumor control probability (TCP) model has been used for estimating the response of the radiation (photon) therapy for a given treatment dose (distribution). In Taiwan, boron neutron capture therapy (BNCT) is still at the stage of the clinical trials without standard dose prescription. In this study, universal survival curve (USC) model was selected as the TCP model for BNCT. The tumor response and dose distribution from protocol I of the clinical trial of the recurrent head and neck (H&N) cancer conducted by Taipei Veterans General Hospital and National Tsing Hua University were used to verify the TCP model established in this study. The results showed that, using the USC model as a biological model of dose conversion, the TCP calculated by the generalized Equivalent Uniform Dose (gEUD)–based TCP model can be used to well correlate the relationship between the tumor response and dose distribution of the patients of recurrent H&N cancer. The result shows that 25% and 60% of TCP correspond to partial response and complete response of H&N cancer, respectively. This study also indicated that, when BNCT was used to treat recurrent H&N cancer, the minimum dose was an important factor on the efficacy of the treatment. Minimum dose of 18 Gy-w corresponds to at least 60% of TCP.

## 1 Introduction

The tumor control probability (TCP) model has been used for estimating the response of the radiation therapy since 1980. More models were developed when more clinical data were collected. In Taiwan, unlike photon therapy, boron neutron capture therapy (BNCT) is still at the stage of the clinical trials without standard prescription dose. The major difference between BNCT and photon therapy is the mechanism of the energy deposited from the interaction. BNCT uses the nuclear reaction of the ^10^B isotope with thermal neutron to produce two high-energy heavy nuclei, alpha (1.84 MeV), and lithium (0.85 MeV), which can release whole kinetic energy in a very short range (10 μm–20 μm). In 2020, boronophenylalanine (BPA), which is a tumor-targeted ^10^B-enriched drug, was approved as a new drug of BNCT in Japan (1). The neutron generator was also approved as a new medical device at the same time (2). In current BNCT protocol, BPA drug is to be injected to the patient for 2h to accumulate high enough ^10^B in the tumor before the neutron irradiation. The ratio of the drug concentration between tumor uptake and normal tissue uptake can be larger than 2.5. The weighted dose (Gy-w) of BNCT is commonly calculated by using the relative biological effectiveness (RBE) of the neutron, 3.2, and compound biological effectiveness (CBE) of the BPA for tumor and normal tissue, 3.8 and 1.3, respectively.

The purpose of this study is to establish a TCP model (3) for BNCT and use a clinical trial result to verify it. The clinical trial result used is the protocol I of the clinical trial (NCT01173172) of the re-current head and neck (H&N) cancer conducted by Taipei Veterans General Hospital and National Tsing Hua University during 2010–2014. It was a two-fraction BNCT treatment protocol. Totally, 17 patients were treated. The prescribed dose for tumor is 80% of tumor volume reaching 20 Gy-w. The principles for the normal tissues and critical organs are to minimize the volumes of the skin and mucosa receiving >10 Gy-w per fraction and limiting the maximum dose of the optic nerve/chiasma to be less than 8 Gy-w per fraction. The efficacy of this clinical trial is 71% (4) of positive response [complete response (CR) and partial response (PR)]. The most common acute adverse events were low-grade oral mucositis, radiation dermatitis, and alopecia. The most common grade 3 late adverse event was cranial neuropathy. No grade 4 late adverse events were observed.

## 2 Materials and methods

### 2.1 Cell survival curve model

In 1976, Douglas and Fowler (5) proposed a linear quadratic (LQ) model to describe the biological effect of conventionally fractionated radiotherapy (CFRT). Cell death by radiation was described by two mechanisms. One is single-strand break, where cell death is proportional to dose-level squared. Another is double-strand break, where cell death is proportional to the dose level. However, according to the large dose protocol used by stereotactic body radiation therapy (SBRT) since 2000, LQ model cannot predict biological effect well for large dose in one fraction. More models were then proposed to correct this.

#### A. Universal survival curve model

Universal survival curve (USC) model was proposed by Park (6) in 2008. This model combines LQ model for low-dose range and multi-target model for high-dose range. The transition dose D_T_ connecting LQ model and multi-target model can be described in Equation 1.


(1) $$\text{lnS}=\{\begin{array}{c} -\left(\text{αd}+\text{β}{\text{d}}^{2}\right)\;\;\text{if}\;\text{d}\leq {\text{D}}_{\text{T}}\\ -\frac{1}{{\text{D}}_{0}}\text{d}+\frac{{\text{D}}_{\text{q}}}{{\text{D}}_{0}}\;\;\;\text{if}\;\text{d}\geq {\text{D}}_{\text{T}} \end{array}$$

where S is survival fraction, d is dose, **α** and **β** are the coefficients, ***D*** *_q_* is the intercept when ***lnS*** is 0 for multi-target model, -1/***D***_0_ is the slope of multi-target model.

#### B. Linear quadratic linear model

Linear quadratic linear (LQL) model was proposed by Astrahan (7) in 2008. It is similar to USC model but with different high dose model as shown in Equation 2.


(2) $$\text{lnS}=\{\begin{array}{c} -\left(\text{αd}+\text{β}{\text{d}}^{2}\right)\;\;\;\;\;\;\;\;\;\;\;\;\;\;\;\;\;\;\text{if}\;\text{d}\leq {\text{D}}_{\text{T}}\\ -\left(\text{α}{\text{D}}_{\text{T}}+\text{β}{\text{D}}_{\text{T}}{\;}^{2}+\text{γ}\left(\text{d}-{\text{D}}_{\text{T}}\right)\right)\;\;\;\text{if}\;\text{d}\geq {\text{D}}_{\text{T}} \end{array}$$

Where ***γ*** is the slope of LQ model at ***D_T_***.

#### C. Padé linear quadratic model

Belkić used Padé approximation to expand LQ model to Padé linear quadratic (PLQ) model (8) in 2013 as shown in Equation 3.


(3) $$\text{lnS}=-\;\frac{\text{αd}+\text{β}{\text{d}}^{2}}{1+\text{γ}\text{d}}$$

**Figure 1:**
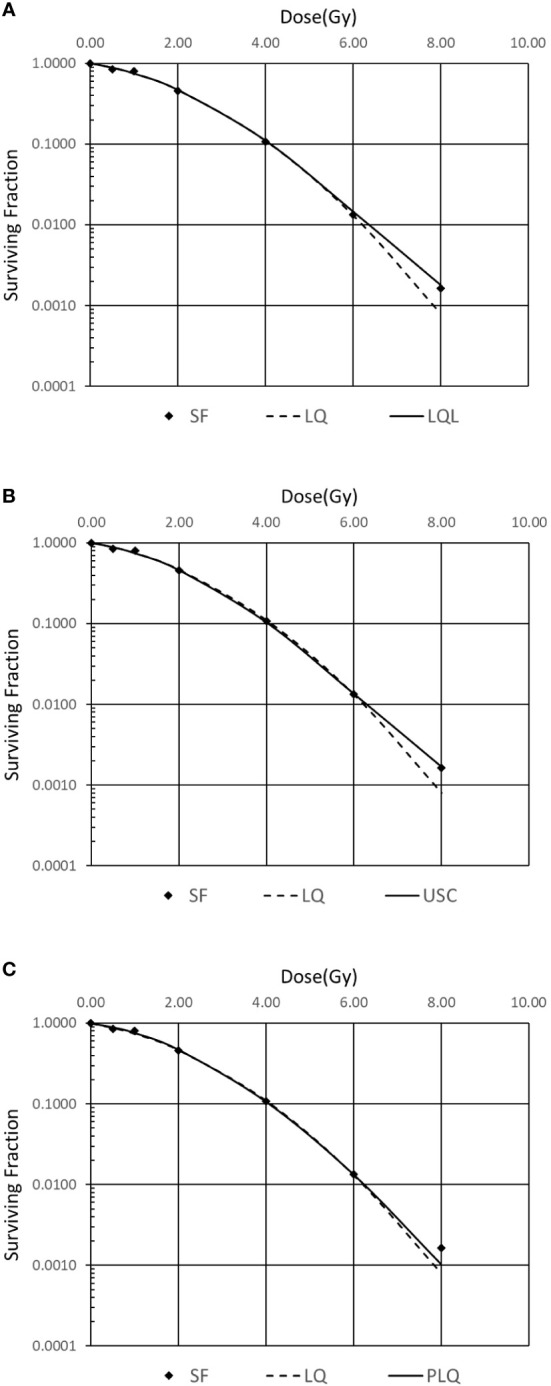
Comparison of different survival curve models for FaDu cell. **(A)** Linear quadratic linear (LQL) model, **(B)** universal survival curve (USC) model, **(C)** Padé linear quadratic (PLQ) model.

In order to investigate which model is suitable for the H&N cancer, the squamous cell carcinoma (SCC) survival fraction data of FaDu cell from Saleh (9) in 2015 is selected. The results are shown in **Figure 1**. Standard error of estimator is used to evaluate the models. The USC model, which has the smallest error, is used in the following step to evaluate biological effectiveness. The coefficients of USC model are listed in **Table 1**.

**Table 1:** Coefficients of USC model for SCC.

Cell type	α (Gy^−1^)	β (Gy^−2^)	D_0_ (Gy)	D_q_ (Gy)	D_T_ (Gy)
SCC	0.2111	0.0890	0.9603	1.8588	4.6628

### 2.2 Biological effective dose

The biological effective dose (BED) represents the dose required for a given biological effect E if the dose were to be delivered by infinitely small doses per fraction. For USC model, the BED can be described by Equation 4 for total dose (*D*) or summation of multiple fractional dose (nd).


(4) BED= Eα=−lnSα={nd(1+dαβ)        if d≤DT1α·D0(nd−n·Dq)   if d≥DT

For single high-dose delivery of BNCT (*D_BNCT_*), which usually is larger than D_T_, BED of BNCT can be described as


(5) $$\text{BED}=\frac{1}{\text{α}\cdot {\text{D}}_{0}}\left({\text{D}}_{\text{BNCT}}-{\;}_{\text{Dq}}\right)$$

Note that *D_BNCT_* is the weighted dose, defined as the sum of the physical dose components multiplied by weighting factors (including RBE and CBE’s). After combining Equations 4 and 5, the equivalent dose in 2 Gy fraction (EQD_2_) for BNCT can be described by Equation 6 when *nd* = EQD_2_ and *d* = 2 Gy:


(6) EQD2(=nd)={1α·D0·DBNCT−Dq(1+2αβ)           if 2 Gy≤DTDBNCT+(n−1)·Dq     if 2 Gy≥DT

### 2.3 Tumor control probability model

According to the logistic formula by Suit (10) in 1965, TCP model with uniform dose (D) in a volume can be described by Equation 7.


(7) $$\text{TCP}\left(\text{D}\right)=\frac{1}{1+{\left(\frac{\text{TC}{\text{D}}_{50}}{\text{D}}\right)}^{4{\text{γ}}_{50}}}$$

where $TCD_{50}$ is the dose for which there is a 50% response, ${\text{γ}}_{50}$ describes the slope of the dose-response curves at the dose of $\;TCD_{50}$ . For SCC, the coefficients are listed below (11).


$$TCD_{50}:\;46.8\;\pm 6.4\text{ Gy}$$

$${\text{γ}}_{50}:2.0$$

Unlike photon therapy, non-uniform dose of tumor must be considered due to the drug and neutron flux distribution in tumor and typical single beam portal for BNCT. Niemierko (12) proposed a generalized Equivalent Uniform Dose (gEUD) as shown in Equation 8,


(8) gEUD=(∑i=1NviDi a)1a=(1N∑i=1NDi a)1a

where $v_{i}$ is fractional volume, *N* is total number of fractional volume, $D_{i}$ is the dose of $v_{i}$ , a is the tissue-specific parameter, for example, −13 for SCC (13). When plugging in gEUD into Equation 7, the EUD-based TCP for non-uniform dose distribution becomes.


(9) $$\text{TCP}\left(\text{gEUD}\right)=\frac{1}{1+{\left(\frac{\text{TC}{\text{D}}_{50}}{\text{gEUD}}\right)}^{4{\text{γ}}_{50}}}$$

Note that, since a of Equation 8 is negative for tumor, gEUD will be mostly affected by the minimum dose of tumor.

There are other existing methods to calculate TCP in addition to generalized EUD-based TCP, for example, subvolumed-based TCP (14). It is found in this study that results of generalized EUD-based TCP, which gives more weight to the minimum tumor dose, correlate well with the clinical results.

Therefore, the procedure for calculating TCP is below:

1) Based on dose-volume histogram (DVH), of BNCT dose report, one can convert $D_{BNCT}$ into equivalent dose in 2 Gy fraction, EQD_2,_ using Equation 6.
2) Because $D_{BNCT}$ in the tumor is non-uniform, one can calculate gEUD using Equation 8 and then obtain TCP using Equation 9.

## 3 Results and discussions

The DVH data of 11 patients with SCC of protocol 1 of BNCT clinical trial for recurrent H&N cancer is used to calculate TCP based on the method developed above. Comparing to the photon therapy, the tumor dose distribution of BNCT is usually non-uniform, especially in large tumors.

The result is shown in **Table 2** together with response of patients and the minimum dose delivered to the tumors during treatment. Although D_BNCT_,_min_ has large weight in determine gEUD [Equation 8], D_BNCT_,_min_ alone cannot predict TCP directly. The whole dose distribution in tumor is needed to calculate gEUD and then TCP. For example, patients 8 and 15 have very close D_BNCT_,_min_, 9.2 and 9.4, respectively, but their gEUD are quite different due to different tumor dose distribution. For patient 8, 15% of tumor dose are below 18.5 Gy-w, while patient 15 have only 2% of tumor dose below 18.5 Gy-w. Therefore, the gEUD of patient 8 is only 31 Gy, while for patient 15 is 44.7 Gy. The resulting TCP of patient 8 is only 4.4, of patient 15 is 40.9. Clinical result shows that patient 8 is stable disease (SD) and patient 15 is PR.

**Table 2:** Results of generalized EUD-based TCP for protocol 1 of clinical trial of recurrent H&N cancer.

Patient no.	D_BNCT,min_ (Gy-w)	gEUD (Gy)	TCP (%)	Response*
1	6.0 (GTV1) 15.3 (GTV2)	21.5 (GTV1) 47.2 (GTV2)	0.2 (GTV1) 51.6 (GTV2)	PD
2	6.5	21.1	0.2	SD
3	10.0	42.3	30.8	PR
4	12.3	41.0	25.9	PR
5	9.2	31.9	4.4	SD
6	19.5	68.6	95.5	CR
7	7.0 (GTV1) 22.3 (GTV2)	22.4 (GTV1) 63.6 (GTV2)	0.3 (GTV1) 92.0 (GTV2)	PD
8	25.3 (GTV1) 19.0 (GTV2)	80.7 (GTV1) 56.5 (GTV2)	98.7 (GTV1) 81.8 (GTV2)	CR
9	18.5	49.8	62.3	CR
10	9.4	44.7	40.9	PR
11	7.9	35.7	10.3	PD

*Accessed using Response Evaluation Criteria in Solid Tumors (RECIST) v 1.1: complete response (CR), partial response (PR), stable disease (SD), and progressive disease (PD).

The results in **Table 2** show that 25% and 60% of TCP correspond to PR and CR of H&N cancer, respectively. When BNCT was used to treat recurrent H&N cancer, the minimum dose was an important factor on the efficacy of the treatment. Minimum tumor dose of 18 Gy-w corresponds to at least 60% of TCP.

## 4 Conclusions

In the radiation therapy field, BNCT is still a new treatment modality and lack of enough clinical data for evaluating the delivered dose related to the patient response. In this study, a BNCT TCP model is established based on the TCP models of photon therapy through the equivalent dose conversion. The TCP calculated using the dose distribution from protocol I of the clinical trial of the recurrent H&N cancer correlates well with the tumor response of the clinical results. The proper TCP range for the prescription dose with good tumor response can be obtained when implementing this model into the BNCT treatment planning system to accumulate more clinical data.

The parameters used in generalized EUD-based TCP model are all based on photon therapy, the cell killing mechanism of which is different from BNCT. More biological data on BNCT is needed.

In future clinical treatment of BNCT, the tumor dose distribution of treatment planning result can be used through this model to calculate TCP to predict outcomes. Preferred treatment plans can then be selected.

## Data availability statement

The data analyzed in this study is subject to the following licenses/restrictions: Clinical trial results involving patients information. Requests to access these datasets should be directed to L-WW, lwwang@vghtpe.gov.tw.

## Ethics statement

Ethical approval was not required for the studies involving humans because this study is a dosimetry research based on a published clinical study, clinical trial [NCT01173172]. Only dose/response data were retrieved. The studies were conducted in accordance with the local legislation and institutional requirements. Written informed consent for participation was not required from the participants or the participants’ legal guardians/next of kin in accordance with the national legislation and institutional requirements because all patients are de-identified. No identifiable human data was shown in this study.

## Author contributions

Conceptualization, F-YH and Y-WHL; methodology, F-YH and Y-WHL; formal analysis, F-YH and Y-WHL; resources, F-YH, Y-WHL, and L-WW (principle investigator of this clinical trial); data curation, F-YH and Y-WHL; writing—original draft preparation, F-YH, Y-WHL, and T-YL; writing—review and editing, Y-WHL and T-YL; visualization, F-YH; supervision, Y-WHL; project administration, Y-WHL; funding acquisition, Y-WHL. Software, F-YH and T-YL. All authors contributed to the article and approved the submitted version.
